# 2314 (In)Adequacy of prophylactic central lymph node dissection for papillary thyroid cancer in the United States: An analysis of 18,755 patients

**DOI:** 10.1017/cts.2018.271

**Published:** 2018-11-21

**Authors:** Keven Seung Yong Ji, Taofik Oyekunle, Julie A. Sosa, Sanziana A. Roman

**Affiliations:** Duke University

## Abstract

OBJECTIVES/SPECIFIC AIMS: The incidence of papillary thyroid cancer (PTC) has sharply increased in recent decades. Though thyroid resection is the best treatment modality, there is significant variation in practice involving use of prophylactic central lymph node dissection (PCLND) at time of thyroidectomy. Recently, a threshold number for lymph node (LN) yield was determined to assure adequacy of lymphadenectomy in evaluating occult nodal disease via PCLND for pathologic T3, clinical N0, M0 PTC patients, for whom guidelines recommend PCLND. This study assesses the prevalence of adequate prophylactic LN dissection (APLND) and determines its association with patient, and disease characteristics. METHODS/STUDY POPULATION: Adult patients receiving surgery for pT3 cN0 M0 PTC >1 cm were identified from the National Cancer Data Base, 2004–2015. APLND for pT3 stage was defined as removing 8 or more LNs, based on recent literature. Univariate and multivariate logistic regression models were employed to determine factors associated with APLND and inadequate prophylactic LN dissection (IPLND). RESULTS/ANTICIPATED RESULTS: In total, 18,755 patients were included: 2905 (10.1%) had APLND; 15,849 (89.9%) had IPLND. Rate of APLND increased from 4.9% to 17.9% over the decade. Patients receiving APLND were younger than those receiving IPLND (47 vs. 52 years, respectively, *p*<0.001). The proportion of cases found to be LN positive in the APLND group was 64.5%, while that in the IPLND group was 18.2% (*p*<0.001). After adjustment, Whites were more likely than Blacks to receive APLND [OR 1.86 (95% CI 1.51–2.30), *p*<0.001]. The adjusted OR of receiving APLND was higher at academic centers [1.76 (1.29–2.41), *p*<0.001] and at integrated centers [1.77 (1.25–2.51), *p*<0.001], compared with community facilities. After adjustment, patients with multifocal tumors were more likely to receive APLND than those with unifocal tumors [1.28 (1.17–1.41), *p*<0.001]. Unplanned 30-day readmission rate was higher in the APLND group (2.4%) compared to the IPLND group (1.7%, *p*<0.001); this remained significant after adjustment [OR for APLND 1.80 (1.31–2.47), *p*<0.001]. There was no significant difference in the likelihood of receiving radioactive iodine between patients who underwent APLND Versus IPLND [1.00 (0.90–1.00), *p*=0.6]. DISCUSSION/SIGNIFICANCE OF IMPACT: APLND is associated with a higher likelihood of finding metastatic LNs, and an increased risk of unplanned short-term readmissions. The rate of APLND has increased over time, but still only a minority of thyroid cancer patients undergo adequate prophylactic surgery. Disparities exist based on patient, facility, and disease characteristics. Further work is needed to study the association between adequacy of dissection and disease recurrence.

